# A computational approach to compare regression modelling strategies in prediction research

**DOI:** 10.1186/s12874-016-0209-0

**Published:** 2016-08-25

**Authors:** Romin Pajouheshnia, Wiebe R. Pestman, Steven Teerenstra, Rolf H. H. Groenwold

**Affiliations:** 1 Julius Center for Health Sciences and Primary Care, University Medical Center Utrecht, PO Box 85500, 3508 GA Utrecht, The Netherlands; 2 Catholic University of Leuven, Research Unit for Quantitative Psychology and Individual Differences, Leuven, Belgium; 3 Scientific Institute for Quality of Healthcare, IQ Healthcare, Radboud University Medical Centre, Nijmegen, The Netherlands; 4 Department for Health Evidence, Section of Biostatistics, Radboud University Medical Centre, Nijmegen, The Netherlands

## Abstract

**Background:**

It is often unclear which approach to fit, assess and adjust a model will yield the most accurate prediction model. We present an extension of an approach for comparing modelling strategies in linear regression to the setting of logistic regression and demonstrate its application in clinical prediction research.

**Methods:**

A framework for comparing logistic regression modelling strategies by their likelihoods was formulated using a wrapper approach. Five different strategies for modelling, including simple shrinkage methods, were compared in four empirical data sets to illustrate the concept of a priori strategy comparison. Simulations were performed in both randomly generated data and empirical data to investigate the influence of data characteristics on strategy performance. We applied the comparison framework in a case study setting. Optimal strategies were selected based on the results of a priori comparisons in a clinical data set and the performance of models built according to each strategy was assessed using the Brier score and calibration plots.

**Results:**

The performance of modelling strategies was highly dependent on the characteristics of the development data in both linear and logistic regression settings. A priori comparisons in four empirical data sets found that no strategy consistently outperformed the others. The percentage of times that a model adjustment strategy outperformed a logistic model ranged from 3.9 to 94.9 %, depending on the strategy and data set. However, in our case study setting the a priori selection of optimal methods did not result in detectable improvement in model performance when assessed in an external data set.

**Conclusion:**

The performance of prediction modelling strategies is a data-dependent process and can be highly variable between data sets within the same clinical domain. A priori strategy comparison can be used to determine an optimal logistic regression modelling strategy for a given data set before selecting a final modelling approach.

**Electronic supplementary material:**

The online version of this article (doi:10.1186/s12874-016-0209-0) contains supplementary material, which is available to authorized users.

## Background

Logistic regression models are frequently utilized in clinical prediction research and have a range of applications [1–4]. While a logistic model may display good performance with respect to its discriminative ability and calibration in the data in which was developed, the performance in external populations can often be much poorer [5–7]. Regression models fitted to a finite sample from a population using methods such as ordinary least squares or maximum likelihood estimation are by nature overfitted and the prediction error can be unacceptably high in new populations [8, 9]. Failure to take this phenomenon into account may lead to poor clinical decision making [10–13], and an appropriate model building strategy must be applied. In the same vein, failure to apply the optimal modelling strategy could also lead to the same problems when the model is applied in clinical practice.

Despite great efforts to present clear guidelines for the prediction model building process [14–16] it may still be unclear to researchers which modelling approach is most likely to yield a model with optimal external performance. At some stages of model development and validation, several approaches could be taken. For example, different forms and combinations of predictors could be modelled, underlying probability distributions could be varied, and penalization could be applied. Each approach may yield a different model, with a different predictive accuracy. Uncertainty over which approach to take may arise even for generally accepted strategies if recommendations are based on simulated or empirical examples that may not be generalizable to the data at hand. In addition, it has been shown that for linear regression the success of a strategy is heavily influenced by a few key data characteristics, and in order to address this a framework was proposed for the a priori comparison of different model building strategies in a given data set [17].

We present an extended framework for comparing strategies in linear and logistic regression model building. A wrapper approach is utilized [18], in which repeated bootstrap resampling of a given data set is used to estimate the relative predictive performance of different modelling strategies. Attention is centred on a single aspect of the model building process, namely, shrinkage-based model adjustment, to illustrate the concept of a priori strategy comparison. We demonstrate applications of the framework in four examples of empirical clinical data, all within the setting of deep vein thrombosis (DVT) diagnostic prediction research. Following from this, simulations highlighting the data-dependent nature of strategy performance are presented. Finally, the outlined comparison framework is applied in a case study, and the impact of a priori strategy selection is investigated.

## Methods

In this section, a framework for the comparison of logistic regression modelling strategies is introduced, followed by a description of the strategies under comparison in this study. The designs of four simulation scenarios using either entirely simulated data or simulated data derived from empirical data are outlined. Finally, the design of a case study in strategy comparison is described. All analyses were performed using the R statistical programme, version 3.1.1 [19]. All computational tools for the comparison of modelling strategies can be found in the “apricom” package, available within the CRAN package repository (http://CRAN.R-project.org/package=apricom).

### A framework for strategy comparison

It was proposed by Pestman et al. [17] that different strategies for linear regression model building could be compared prior to selecting a final strategy by means of a simple framework. The predictive performance of a linear regression model in a data set can be summarized by the sum of squared errors (SSE) [20]. In order to compare two different models, A and B, the SSE of each model could be compared directly by taking the ratio SSE(B)/SSE(A). A ratio greater than 1 indicates the SSE of B is greater than that of A, and therefore model B has a poorer predictive performance.

**Fig. 1:**
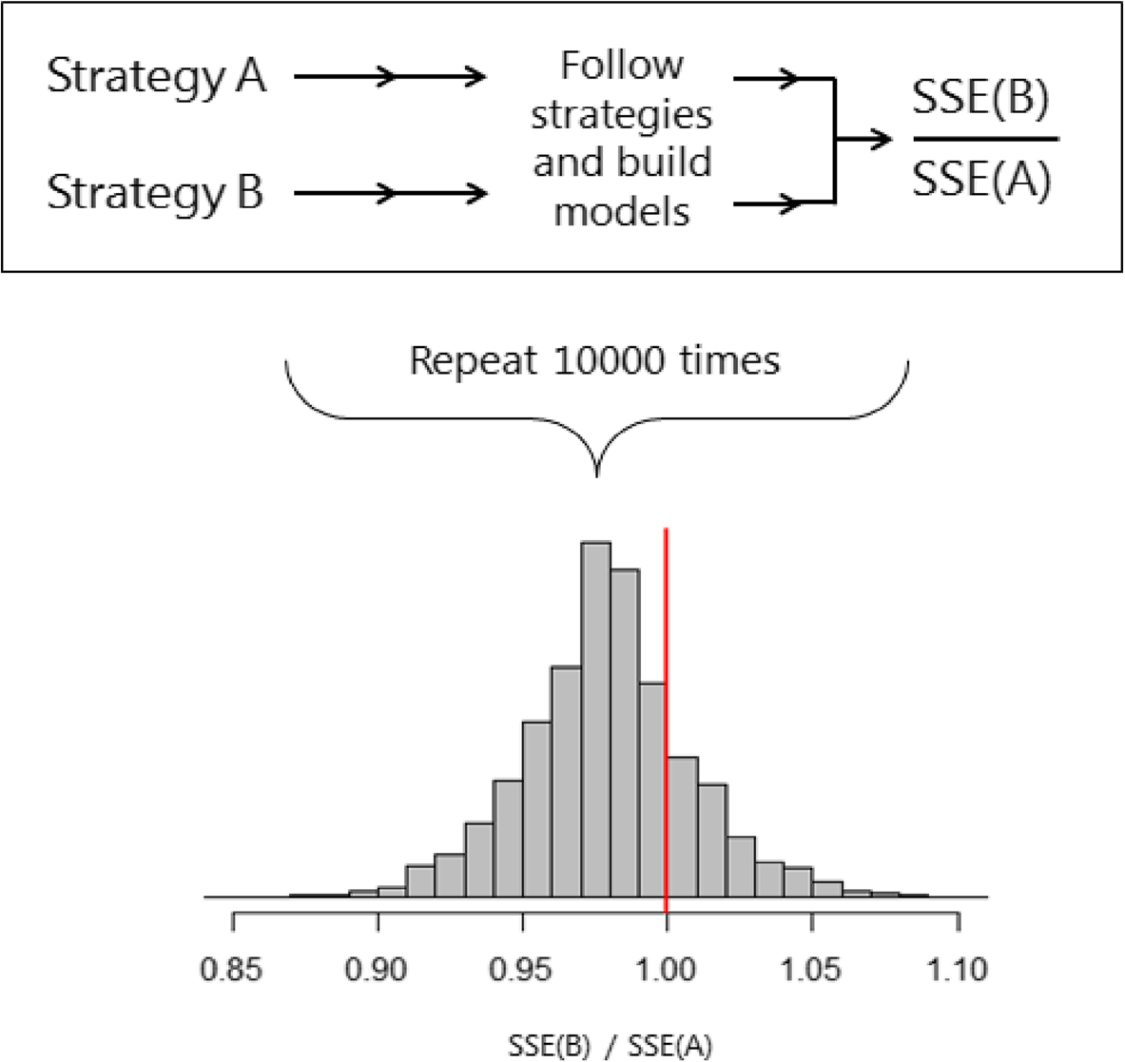
An example of the comparison of two linear regression modelling strategies. Strategies A and B are individually applied to a data set and the ratio SSE(B)/SSE(A) is calculated. The process is repeated 10,000 times yielding a comparison distribution. The left tail below a cut off value of 1 represents the victory rate of strategy B over strategy A, the proportion of times strategy B outperformed strategy A

This concept can in theory be extended to the comparison of different modelling strategies. However, aspects of modelling that involve sampling or data splitting have a random element, and repetition of the comparison would give different results each time. In order to obtain a general comparison of two strategies, the process of model building and SSE estimation could be repeated many times, each time yielding a different ratio of the SSEs. This will eventually produce a distribution of SSE ratios. This distribution can be used to make inferences about the performance of one modelling strategy compared to another in a given set of data. One useful measure is the proportion of times that the ratio SSE(B)/SSE(A) is less than 1, which has previously been referred to as the “victory rate” (VR). This estimates the probability that a model built using strategy B will outperform a model built using strategy A. An example of the overall concept of strategy comparison, and the kind of distribution it yields is illustrated in Fig. 1.

While the SSE can be used to compare the performance of two linear models, it cannot be readily extended to the setting of logistic regression. The log likelihood is a commonly used measure to assess the fit of a logistic regression model [16]. Nested models can be compared by taking the ratio of the likelihoods of the models. The difference in -2 log likelihoods of models built using two different strategies will yield a distribution of log-ratios when subjected to repeated sampling. The proportion of times the log-ratio falls below zero estimates the probability that strategy B will outperform strategy A in the given data.

In addition to the victory rate, the comparison distribution, consisting of SSE ratios or differences in -2 log likelihoods, can be characterized by looking at its median value and interquartile range. This gives an indication of the magnitude and variability of the difference in performance of the two strategies under comparison. It may be the case that the victory rate of one strategy over another approaches 100 %, implying that it is the superior choice. However, if the median value is very close to 1 for linear regression or 0 for logistic regression, then the absolute differences in performance could be considered so small that the strategies are equally good.

For the analyses in this study, we implemented the concept shown in Fig. 1 within a resampling framework. Bootstrapping was used to repeatedly generate samples from an initial data set, and a model was fitted in each bootstrap sample according to each strategy. The models were then applied in the initial data set, which can be seen to represent the “true” source population, and the model likelihood or SSE was estimated.

### Shrinkage and penalization strategies

In this study, six different modelling strategies were considered. The first strategy, which was taken as a common comparator for the others, is the development of a model using either ordinary least squares or maximum likelihood estimation, for linear and logistic regression respectively, where predictors and their functional forms were specified prior to modelling. This will be referred to as the “null” strategy. Models built following this strategy often do not perform well in external data due to the phenomenon of overfitting, resulting in overoptimistic predictions [8, 9, 21, 22]. The remaining five strategies involve methods to correct for overfitting.

Four strategies involve the application of shrinkage techniques to uniformly shrink regression coefficients after they are estimated by ordinary least squares or maximum likelihood estimation. Strategy 2, which we will refer to as “heuristic shrinkage”, estimates a shrinkage factor using the formula derived by Van Houwelingen and Le Cessie [23]. Regression coefficients are multiplied by the shrinkage factor and the intercept is re-estimated [14]. Strategies 3, 4 and 5 each use computational approaches to derive a shrinkage factor [5, 7, 24]. For strategy 3, the data set is randomly split into two sets; a model is fitted to one set, and this model is then applied to the other set in order to estimate a shrinkage factor. Strategy 4 instead uses k-fold cross-validation, where k is the number of subsets into which the data is divided, and for each of the repeats of the cross-validation, a model is fitted to k-1 subsets and applied to the remaining set to derive a shrinkage factor. Strategy 5 is based on resampling and a model is fitted to a bootstrap replicate of the data, which is then applied to the original data in order to estimate a shrinkage factor. These methods will be referred to as “split-sample shrinkage”, “cross-validation shrinkage” and “bootstrap shrinkage” respectively.

The final strategy uses a form of penalized logistic regression [25, 26]. This is intrinsically different to the approaches described above. Instead of estimating a shrinkage factor and applying this uniformly to the estimated regression coefficients, shrinkage is applied during the coefficient estimation process in an iterative process, using a Bayesian prior related to Fisher’s information matrix. This method, which we will refer to as “Firth penalization”, is especially appealing in sparse data settings with few events and many predictors in the model.

### Clinical data sets

A total of four data sets, each consisting of data used for the prediction of deep vein thrombosis (DVT) were used in our analyses.

Set 1 (“Full Oudega”) consists of data from a cross-sectional study of 1295 adult patients suspected of having DVT, collected from 1^st^ January 2002 to June 1^st^ 2003, within a primary care setting in the Netherlands, having gained approval from The Medical Research Ethics Committee of the University Medical Center Utrecht [27]. Information on 26 potential predictors of DVT presence was collected, and a prediction rule including 8 dichotomous predictors was developed using logistic regression.

Set 2 (“Oudega subset”) was derived by taking a sample of 500 observations, without replacement, from set 1. The resulting data has a similar case mix, but the total number of outcome events was reduced from 289 to 110.

Set 3 (“Toll validation”) was originally collected as a data set for the temporal validation of set 1. Data from 791 patients with suspected DVT was collected in the same manner as set 1, but from 1^st^ June 2003 to 1^st^ January 2006, after the collection of the development data [28, 29]. This data set contains the same predictors as sets 1 and 2.

Set 4 (“Deepvein”) consists of partly simulated data available from the R package “shrink” [30]. The data are a modification of data collected in a prospective cohort study of 929 patients between July 1992 and August 2008, from four centres in Vienna, Austria [31]. As this data set comes from a completely different source to the other three sets, it contains different predictor information. Furthermore, a combination of continuous and dichotomous predictors was measured.

Data set 4 can be accessed in full via the R programming language “shrink” package. Data sets 1–3 are not openly available, but summary information for the data sets can be found in Additional file 1, which can be used to simulate data for reproduction of the following analyses.

### Strategy comparison in clinical data

Strategies for logistic regression modelling were first compared using the framework outlined in 2.1, in the Full Oudega data set, with 5000 replicates for each comparison. For each strategy under comparison, full logistic regression models containing all 8 available predictors were fitted. The shrinkage and penalization strategies were applied as described in 2.2. For the split sample strategy, data was split so that the initial model fitting was done in 80 % of the data, and the process was repeated 100 times for stability. For the cross-validation strategy, 10-fold cross-validation was performed, and averaged over 10 replicates. For the bootstrap strategy, 100 rounds of bootstrapping were performed. For the final strategy, Firth regression was performed using the “logistf” package, in the R programming language [32]. These strategies were then compared against the null strategy, and the distributions of the differences in -2 log likelihoods over all comparison replicates were plotted as histograms. Victory rates, distribution medians and distribution interquartile ranges were calculated from the comparison results. The mean shrinkage was also calculated where appropriate.

### Simulations

To investigate the extent to which strategy performance may be data-specific, simulations were performed to compare the performance of the modelling strategies from 2.2 across ranges of different data parameters. To compare strategies in linear regression modelling, data were entirely simulated, using Cholesky decomposition [33], and in all cases simulated variables followed a random normal distribution with mean equal to 0 and standard deviation equal to 1. In each scenario the number of predictor variables was fixed at 20. Data were generated so that the “population” data were known, with 1000 observations. In scenario 1, the number of observations per variable in the model (OPV) was varied by reducing the number of rows in the data set in increments from 500 to 50, whilst maintaining a model R^2^ of 0.5. In scenario 2, the fraction of explained variance, summarized by the model R^2^, was varied from 0.1 to 0.9, whilst the OPV was fixed at a value of 5. For each linear regression setting, comparisons were repeated 10,000 times.

To compare strategies in logistic regression modelling, the full Oudega data set and Deepvein data set were used. In scenario 3, the number of outcome events per model variable (EPV) was varied by removing cases and non-cases from the data incrementally, resulting in EPVs ranging from 36 to 4, whilst maintaining a similar case-mix and prevalence of DVT. This was also repeated in the Deepvein data, with values for the EPV ranging from 24 down to 4. In scenario 4, strategies were compared in the full Oudega data across a range of settings where the fraction of explained variance, taken to be the value of Nagelkerke’s R^2^ [34], varied. First, a subset of 15 dichotomous variables was selected from the total of 26 available variables. Then, selecting 8 variables at a time, the data was sampled in order to generate a large number of subsets, each containing different combinations of predictors, and from these a selection of data sets was chosen based on the Nagelkerke R^2^ of a logistic model fitted to that data, so that a range of Nagelkerke R^2^ values would be covered. For logistic regression scenarios, simulations were repeated 5000 times due to the greater computation time.

### Clinical case study

A small case study was conducted in order to assess whether an a priori comparison of strategies for developing a regression model will provide a model that performs best in external data. Final models were developed in the full Oudega set using the winning strategies from 2.4, as well the null strategy as a reference. In order to directly assess the performance of a given strategy the external predictive performance of each model was assessed in the Toll validation data. The predictive accuracy of each model developed according to each strategy was measured by calculating the Brier score [35], a function of the mean squared prediction error. Calibration of the model was assessed graphically by plotting predicted risks, grouped in deciles, against the observed outcome rates in each decile, using the R package “PredictABEL” [36].

## Results

### Strategy comparison in four clinical data sets

Table 1 shows the results of the comparisons for all five strategies against the null strategy, in the full Oudega data. Firth penalized regression (66.9 %), split-sample shrinkage (66.8 %) and bootstrap shrinkage (66.4 %) had the highest victory rates. The bootstrap shrinkage strategy had the distribution median furthest from zero (-0.3), and a relatively large interquartile range (1.0), indicating possible superiority in this setting, as well as inconsistency.

**Table 1:** A comparison of modelling strategies against the null strategy in the full Oudega DVT data

Strategy	Victory rate (%)	Median	IQR	Mean shrinkage
1. Heuristic shrinkage	56.9	−0.2	1.5	0.97
2. Split sample shrinkage	66.8	−0.2	0.7	0.98
3. 10-fold CV shrinkage	48.0	0.0	0.1	1.00
4. Bootstrap shrinkage	66.4	−0.3	1.0	0.97
5. Firth penalization	66.9	−0.2	0.6	-*

Victory rates and associated metrics are presented. Values are based on 5000 comparison replicates. *Abbreviations*: *IQR* interquartile range, *CV* cross-validation

*No mean shrinkage for the Firth penalization strategy is presented as shrinkage occurs during the coefficient estimation process

**Fig. 2:**
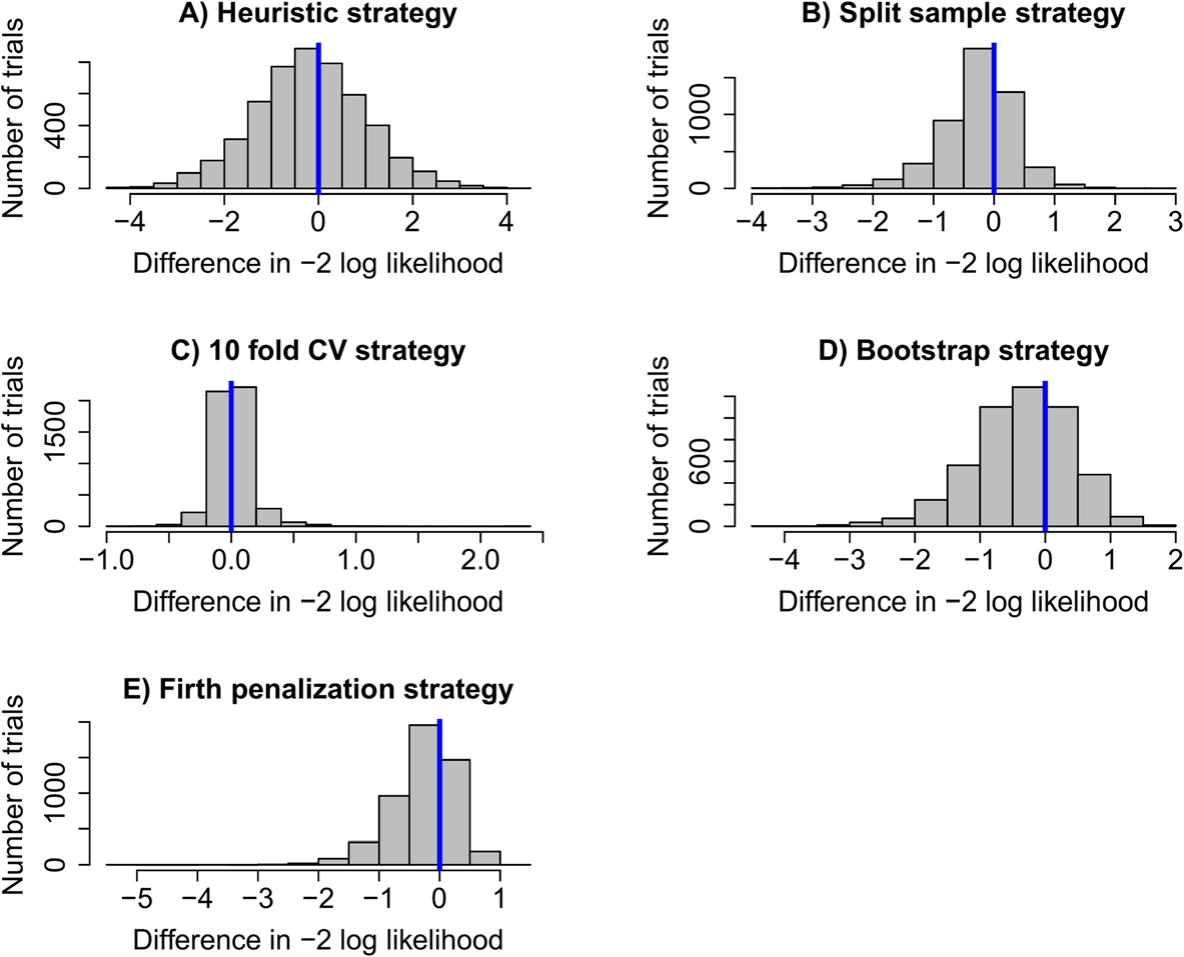
Histograms of the distributions resulting from comparisons between five modelling strategies and the null strategy in the full Oudega data set. The victory rate of each strategy over the null strategy is represented by the proportion of trials to the left of the blue indicator line. The distributions each represent 5000 comparison replicates

The distributions in Fig. 2 indicate that none of the strategies showed a clear superiority over the null strategy in the full Oudega data. For the Firth penalized regression strategy, the distribution is left-skewed, indicating that in some of the comparison replicates this strategy greatly outperformed the null strategy. Given these results, the Firth strategy might be considered to be the optimal choice, as it has both an equally high chance of outperforming the null strategy as compared to the split-sample and bootstrap approaches, and in trials where it had a poorer performance, the difference in -2 log likelihoods was minimal.

When comparisons were extended to additional DVT prediction data sets, a large degree of heterogeneity was observed in the victory rates for each strategy across the different sets. The results of these comparisons are summarized in Table 2. The victory rates of the heuristic strategy showed the greatest variation between data sets, ranging from 3.9 to 63.8 %. This is reflected by the broad range in values of the estimated shrinkage factor, with poorest performance coinciding with severe shrinkage of the regression coefficients. Firth regression showed the greatest consistency between data sets, with victory rates ranging from 65.8 to 73.8 %, and good performance in the Oudega and Toll data sets, but relatively poor performance compared to the split-sample, cross-validation and bootstrap strategies in the Deepvein data set.

**Table 2:** A comparison of modelling strategies in three additional clinical data sets

Strategy	*Oudega random subset*		*Toll validation data set*		*Deepvein data set*	
	Victory rate (%)	Mean shrinkage	Victory rate (%)	Mean shrinkage	Victory rate (%)	Mean shrinkage
1. Heuristic shrinkage	63.8	0.93	60.8	0.93	3.9	0.71
2. Split sample shrinkage	61.9	0.92	42.0	0.94	93.8	0.98
3. 10 fold CV shrinkage	38.3	1.00	39.6	0.99	90.9	0.99
4. Bootstrap shrinkage	56.4	0.89	42.6	0.94	94.9	0.97
5. Firth penalization	73.8	-*	66.0	-*	65.8	-*

Victory rates of each strategy over the null strategy are presented, as well as the mean shrinkage factor applied in each of the shrinkage-based strategies. Values are based on 5000 comparison replicates. *Abbreviations*: *CV* cross-validation

*No mean shrinkage for the Firth penalization strategy is presented as shrinkage occurs during the coefficient estimation process

### Simulation study

**Fig. 3:**
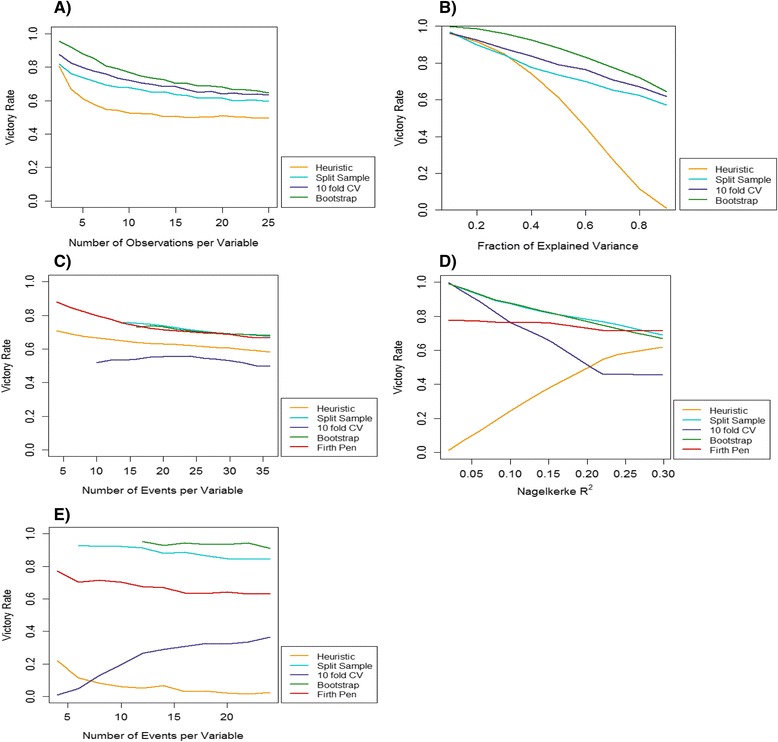
**a**-**e** The influence of data characteristics on the performance of different modelling strategies compared to the null strategy. Victory rates were estimated across a range of values of a data parameter, keeping all other parameters fixed. **a** Linear regression using simulated data; the number of observations in the data per model variable was varied. **b** Linear regression using simulated data; the fraction of explained variance (R^2^) of the least squares model was varied. **c** Logistic regression using simulated data based on the full Oudega data; the number of outcome events in the data per model variable was varied. **d** Logistic regression using simulated data based on the full Oudega data; the explained variance (Nagelkerke’s R^2^) of the maximum likelihood model was varied. **e** Logistic regression using simulated data based on the Deepvein data; the number of outcome events in the data per model variable was varied. * A loess smoother was applied to (**c**), (**d**) and (**e**)

Figure 3a shows that for each strategy, the victory rate decreased as the OPV increased, and the relationship was most apparent when the OPV was less than 10. Similarly, Fig. 3b shows that as the explanatory power of the predictors in the model increased, leading to an increase in the model R^2^, the victory rates for each strategy decreased. However, not all strategies behaved similarly, for example, as the fraction of explained variance increased above 0.4, the performance of the heuristic approach declined drastically.

The performance of logistic regression modelling strategies was also dependent on the information in a data set. Figure 3c shows that in the full Oudega data set, the victory rates of shrinkage strategies declined slightly as the EPV increased, however estimation of the victory rates in low EPV settings was not always possible for the split-sample, cross-validation and bootstrap strategies. The fraction of explained variance of the model had a greater influence on strategy performance. Figure 3d shows that while most strategies show a general decline in performance as the model Nagelkerke R^2^ increases, the heuristic approach improves drastically, from almost zero, to over 50 % across the parameter range.

Comparing Fig. 3c and e highlights that the relationship between strategy performance and a single data characteristic may vary between data sets. While most strategies showed a similar decline in performance as the EPV increased, in the Deepvein data 10-fold cross-validation began to improve as the EPV increased, and both 10-fold cross-validation and the heuristic approach performed very poorly in all EPV settings.

### Case study

Based on the victory rates and distribution medians from Table 1, and assessment of the graphs in Fig. 2, three potentially optimal strategies were selected: the split-sample approach, the bootstrap approach and the Firth regression approach. Differences between these methods were so small that no clear preference could be made between the three.

The winning strategies and the null strategy were applied to the full Oudega data and the resulting models can be found in Additional file 2. Application of the newly developed models to the Toll validation data found that the Brier scores for each model were almost identical, ranging from 0.125 to 0.126 and there was almost no difference in calibration. Calibration plots can be found in Additional file 3. This indicates that in this setting a priori strategy selection has little impact on the external performance of the final model.

## Discussion

There are numerous approaches for developing a clinical prediction model, and in many cases no approach is universally superior. We demonstrate here that the performance of regression modelling strategies is data set-specific, and influenced by a combination of different data characteristics. We outline a means of conducting a comparison of modelling strategies in a data set before deciding on a final approach. A concept that was previously outlined for linear regression has now been extended to logistic regression, using the model likelihood as a means of comparing the performance of two strategies. The resulting distribution of comparisons can then provide researchers with evidence on which to base their decisions for model building. Three summary measures, the victory rate, the distribution median and the distribution interquartile range can be used to guide researchers in their analytical decision making.

As there are several available strategies for addressing the issue of overfitting, we used this as an example to illustrate how different strategies can be compared in a given data set using a computational framework. This article makes no recommendations for which shrinkage approach is more suitable than others; on the contrary our findings highlight that the optimal approach for model building using shrinkage or penalization largely depends on the data at hand, and it can be difficult to anticipate beforehand how well a method is likely to perform.

The comparisons that we conducted in empirical data clearly show that strategy performance is inconsistent and hard to predict across data sets. This is evidenced by the variability in the victory rates presented in Tables 1 and 2. Despite having a very similar case-mix, the victory rates of shrinkage strategies over the null strategy varied by almost 25 % across the three related DVT data sets. These differences between the different data sets may be partly explained by differences in outcome prevalence and the dichotomization of predictors. A detailed discussion of the performance and properties of shrinkage approaches is beyond the scope of this article and can be found elsewhere [7, 15, 37, 38]. Using the results of these comparisons, it is possible to select a winning strategy for each individual data set. However, it is not sufficient to base decisions for model building solely on the victory rate. For example, the victory rate of 90.9 % for 10-fold cross-validation in the Deepvein data set, shown in Table 2, suggests that this strategy is preferable to a strategy without shrinkage. However, the absolute amount of shrinkage being performed is on average negligible in this case, and the high victory rate for cross-validation reflects very small improvements in model performance. We therefore recommend that the median and shape of the comparison distribution should also be taken into account when using this approach for strategy selection.

In some settings, particularly the Oudega subset and Toll data, we observed problems with model convergence in logistic regression due to separation [39]. This problem was most apparent in data with only dichotomous variables in the models, and few EPV. The drop in victory rates for sampling-based strategies, from 66.8 to 61.9 % for sample splitting, 48.0 to 38.3 % for 10 fold cross-validation, and 66.4 to 56.4 % for bootstrapping could in part be explained by this phenomenon. We found that some strategies may exacerbate problems with separation, and that low victory rates, with extremely skewed comparison distributions may indicate the occurrence of separation. In such a case, researchers may wish to consider alternative strategies.

Several authors have previously noted that regression methods may perform quite differently according to certain data parameters [7, 40], and has been recognized that data structure as a whole should be considered during model building [41]. Our simulations in linear regression confirm the findings of others in a tightly controlled setting, and similar trends are seen upon extending these simulations to empirically-derived settings for logistic regression. Through assessing the influence of EPV on strategy performance in two different data sets, we find that while trends are present, they may differ between data sets. In combination with the findings from comparisons between strategies in four clinical data sets this supports the idea that strategy performance is data-dependent. This may have implications for the generalizability of currently existing recommendations for several stages of the model building process that were originally based on a small number of clinical examples.

The findings of our case study did not demonstrate any clear benefit of a priori strategy comparison. This can be explained in part by the similarity of the models produced using each approach, seen in Additional file 2, due to the minimal amount of shrinkage that was applied, and the similarity between the development and validation data. A greater benefit may be expected if the shrinkage strategies were applied first in a data set that would be more susceptible to overfitting, and if the validation data came from a wholly unrelated population. These findings also demonstrate the probabilistic nature of our comparisons. For example, the victory rate of 66.4 % for the bootstrap approach, shown in Table 1, implies that one third of the time maximum likelihood models developed in similar samples from this population of DVT patients will outperform models built using bootstrap-derived shrinkage. Therefore, it is essential to note that a priori strategy comparison may have a limited impact in some settings.

Our study provides a unique approach to decision making in regression model building for clinical prediction research. While similar approaches are used in other fields, they have not been adopted in clinical research and merit further investigation. In extending previous methodology for linear regression strategy comparison to the setting of logistic regression, our findings now have a much greater relevance to clinical research. We also suggest ways to interpret the results of strategy comparisons, providing summary measures and graphical displays that can be used in combination to select a strategy. Furthermore, we used multiple data sets to illustrate how a priori strategy comparison can be applied in practice, and provide open access tools in the R programming language for other researchers to further explore the comparison framework and apply it to their own research.

It must be noted that there are limitations within the current framework. Our study only focuses on comparisons within the domain of shrinkage, which is only one stage of the prediction modelling process. It may be that our approach is not suitable for certain aspects of model building that we have not explored. For example, strategies that yield models that use varying numbers of degrees of freedom should not be compared directly by their model likelihoods. Furthermore, we currently only provide a framework for linear and logistic regression problems, and while this is most useful for diagnostic settings, a natural extension would be to enable the comparison of survival models, such as Cox proportional hazards models, as these are the most commonly used methods in prognostic prediction modelling [42].

Furthermore, the interpretation of the results of comparisons warrants some caution when using logistic regression in sparse data settings. We encountered many difficulties with separation of logistic regression, especially when resampling or sample-splitting methods were used in the model building process. When separation occurs, the models may exhibit problems with convergence, and this complicates the interpretation of victory rates and other summary measures. While there is no straightforward solution to this problem, we argue that there may be some value in observing the frequency and severity of separation that occurs during strategy comparison.

## Conclusion

Current literature provides several guidelines to aid researchers in selecting an appropriate strategy for clinical prediction modelling. Our findings highlight an insufficiency in such approaches due to the influence of data-specific properties on the performance of modelling strategies. A wrapper approach can be used to compare different strategies prior to the final model building process. Optimal strategies can then be selected based on a combination of comparison metrics, but the impact of this approach on the final external performance of a model may be limited.

## Additional files

Additional file 1: Data summary table- A summary of the predictor information in the four DVT data sets. (DOCX 15 kb) Additional file 2: Regression coefficient table- Regression coefficients for the maximum likelihood model and the models built in accordance with the three winning strategies. (DOCX 15 kb) Additional file 3: Calibration plots- Calibration plots of models developed in the Full Oudega data using the winning strategies, assessed in the Toll validation data. (DOCX 188 kb)

## Abbreviations

DVT: Deep vein thrombosis
SSE: Sum of squared errors
VR: Victory rate
OPV: Number of observations per model variable
EPV: Number of outcome events per model variable
IQR: Interquartile range
CV: Cross-validation
