# Supplementary material for: A computational approach to compare regression modelling strategies in prediction research
Source: BMC Med Res Methodol. 2016 Aug 25;16(1):107. doi: 10.1186/s12874-016-0209-0 (PMC4997720; doi:10.1186/s12874-016-0209-0)
Supplement: Additional file 1: — Data summary table- A summary of the predictor information in the four DVT data sets. (DOCX 15 kb) [file 12874_2016_209_MOESM1_ESM.docx]

Additional File 1

Table: A summary of the predictor information in the four DVT data sets. Means or proportions are reported with standard deviations in parentheses.

| Variable | Full Oudega  n = 1295 | Oudega subset  n = 500 | Toll validation  n = 791 | Deepvein  n = 929 |
| --- | --- | --- | --- | --- |
| DVT (outcome) | 0.22 (0.42) | 0.22 (0.41) | 0.16 (0.37) | 0.16 (0.37) |
| Sex | 0.36 (0.48) | 0.36 (0.48) | 0.38 (0.49) | 0.40 (0.49) |
| No trauma | 0.85 (0.36) | 0.85 (0.36) | 0.82 (0.38) | - |
| Oral contraceptive use | 0.10 (0.30) | 0.10 (0.30) | 0.10 (0.30) | - |
| Malignancy | 0.06 (0.24) | 0.08 (0.27) | 0.05 (0.21) | - |
| Surgery | 0.14 (0.25) | 0.14 (0.35) | 0.13 (0.34) | - |
| Vein distension | 0.20 (0.40) | 0.19 (0.39) | 0.20 (0.40) | - |
| Calf difference > 3cm | 0.43 (0.50) | 0.45 (0.50) | 0.41 (0.49) | - |
| Log D-dimer (dichotomous) | 0.69 (0.46) | 0.71 (0.46) | 0.72 (0.45) | - |
| Log D-dimer (continuous) | - | - | - | 8.53 (1.01) |
| Factor II mutation | - | - | - | 0.95 (0.21) |
| Factor V Leiden | - | - | - | 0.77 (0.42) |
| Body mass index | - | - | - | 27.97 (4.46) |
| Age | - | - | - | 54.00 (13.09) |
| Duration of anti- coagulants | - | - | - | 7.65 (3.30) |
| Location of first thrombosis: | - | - | - | distal: 0.18  pulmonary: 0.47  proximal: 0.35 |

* Abbreviations: DVT, deep vein thrombosis
