# Supplementary material for: A computational approach to compare regression modelling strategies in prediction research
Source: BMC Med Res Methodol. 2016 Aug 25;16(1):107. doi: 10.1186/s12874-016-0209-0 (PMC4997720; doi:10.1186/s12874-016-0209-0)
Supplement: Additional file 2: — Regression coefficient table- Regression coefficients for the maximum likelihood model and the models built in accordance with the three winning strategies. (DOCX 15 kb) [file 12874_2016_209_MOESM2_ESM.docx]

Additional File 2

Table: Regression coefficients for the maximum likelihood model and the models built in accordance with the three winning strategies.

| Strategy | Int. | Gender | No trauma | OC. use | Malign. | Surgery | Vein dist. | Log D-dimer | Calf diff. > 3 cm | |
| --- | --- | --- | --- | --- | --- | --- | --- | --- | --- | --- |
| 1. Maximum  likelihood* | -5.024 | 0.708 | 0.668 | 0.758 | 0.500 | 0.418 | 0.534 | 2.432 | 1.145 |  |
| 2. Split sample  shrinkage | -4.939 | 0.694 | 0.654 | 0.743 | 0.489 | 0.410 | 0.523 | 2.382 | 1.122 |  |
| 3. Bootstrap  shrinkage | -4.884 | 0.684 | 0.645 | 0.733 | 0.483 | 0.404 | 0.516 | 2.350 | 1.106 |  |
| 4. Firth  penalization | -4.949 | 0.702 | 0.653 | 0.758 | 0.499 | 0.417 | 0.531 | 2.386 | 1.134 |  |

* Abbreviations: Int, intercept; OC, oral contraceptive; Malign, malignancy; dist, distension; diff, difference.

***** This corresponds to the null strategy in which no shrinkage or penalization was performed.
