# Supplementary material for: A computational approach to compare regression modelling strategies in prediction research
Source: BMC Med Res Methodol. 2016 Aug 25;16(1):107. doi: 10.1186/s12874-016-0209-0 (PMC4997720; doi:10.1186/s12874-016-0209-0)

Additional File 3

Figure: Calibration plots of models developed in the Full Oudega data using the winning strategies, assessed in the Toll validation data.


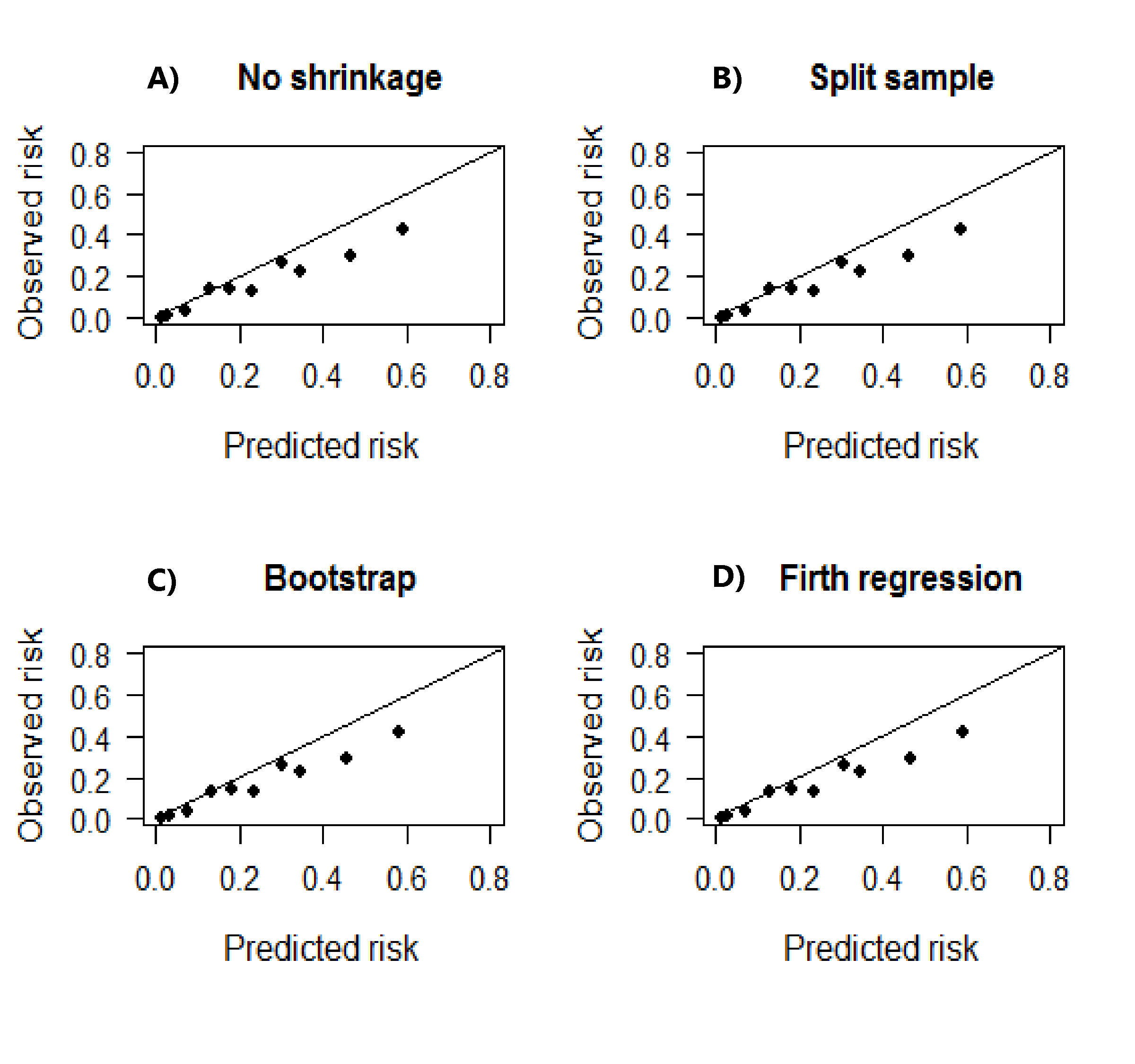

Supplement: Additional file 3: — Calibration plots- Calibration plots of models developed in the Full Oudega data using the winning strategies, assessed in the Toll validation data. (DOCX 188 kb) [file 12874_2016_209_MOESM3_ESM.docx]
